# The Clinical Course of the Late Toxicity of Definitive Radiotherapy in Cervical Cancer

**DOI:** 10.3390/medicina60081364

**Published:** 2024-08-21

**Authors:** So Jung Lee, Myungsoo Kim, Yoo-Kang Kwak, Hye Jin Kang

**Affiliations:** Department of Radiation Oncology, Incheon St. Mary’s Hospital, College of Medicine, The Catholic University of Korea, Seoul 06591, Republic of Korea; mskim0710@gmail.com (M.K.); behappy1219@catholic.ac.kr (Y.-K.K.); kanghj@catholic.ac.kr (H.J.K.)

**Keywords:** cervix cancer, onset, prevalence, late toxicity, radiotherapy

## Abstract

*Background and Objectives*: This study aimed to investigate the clinical course and characteristics of late toxicity over time following the completion of definitive radiotherapy (RT) in patients with cervical cancer. *Materials and Methods*: We retrospectively reviewed the medical records of 60 patients with cervical cancer who underwent pelvic external beam radiotherapy followed by intracavitary brachytherapy. Late toxicity was assessed for the lower gastrointestinal (GI) tract and bladder organ at 6, 12, 24, 36, and >36 months post-RT. We examined the onset and prevalence of late toxicity at each time point. Clinical remission and interventions for managing late toxicity were also investigated. *Results*: The peak onset of lower GI toxicity occurred 12 months after RT completion, with a median symptom duration of 9.9 months (range, 0.1–26.3 months), and exhibited its highest prevalence rate of 15.5% at 24 months post-RT. Most GI toxicities developed and resolved within three years post-RT, with a prevalence rate of 8.1% at three years, followed by a decreasing trend. Bladder toxicity first peaked at 24 months post-RT and continued to occur beyond 36 months, showing the re-increasing pattern in the prevalence rate after 36 months (23.5%). In terms of clinical remission, 66.7% of lower GI toxicities (12 of 18 patients) and 60% of bladder toxicities (9 of 15 patients) achieved complete remission by the last follow-up date. *Conclusions*: Late toxicities of the GI and bladder following definitive RT in cervical cancer are partially reversible and exhibit distinct patterns of onset and prevalence over time. A systematic follow-up strategy should be established for the early detection and timely intervention of late toxicity by understanding these clinical courses.

## 1. Introduction

Cervical cancer is the fourth most common malignant tumor among women, with an estimated 604,000 new cases diagnosed annually worldwide [1]. Radiation therapy (RT) plays a crucial role in treating advanced cervical cancer. The National Comprehensive Cancer Network guidelines classify patients with stage IB2, IIA2, and IIB–IVA as having advanced cervical cancer and recommend concurrent chemoradiotherapy (CCRT) followed by brachytherapy [2]. In definitive RT for cervical cancer, the prescribed high-dose radiation poses a risk of long-term toxicity, which can diminish a patient’s quality of life and may require medical interventions or surgery [3,4]. Although numerous studies have reported on the incidence rates of late toxicity [5,6,7,8,9], there is limited research concerning the clinical course of late toxicity over time. Incidence rates provide an estimate of the maximum toxicity observed at a specific time point within a given period, so this method has limitations in understanding the clinical course over time of toxicity. This study aims to investigate the clinical course and characteristics of late toxicity over time in the lower gastrointestinal (GI) tract and bladder by analyzing the onset and prevalence at various time points after completing RT. Additionally, we aim to assess the clinical remission of toxicity and the effectiveness of interventions.

## 2. Materials and Methods

### 2.1. Patients

We retrospectively reviewed the medical records of patients diagnosed with 2009 International Federation of Gynecology and Obstetrics (FIGO) stage IB-IVA cervical cancer who received RT with curative intent between January 2006 and June 2023. Of the 90 patients who underwent definitive RT, exclusions were made as follows: 23 patients who received an intensity-modulated radiation therapy (IMRT) boost instead of intracavitary brachytherapy (ICBT), 1 patient with a history of prior pelvic irradiation, 2 patients who did not complete RT, and 4 patients who were lost to follow-up. Ultimately, 60 patients who received external beam radiation therapy (EBRT) with or without concomitant chemotherapy followed by ICBT were included in this study.

### 2.2. Treatments

A total of 7 patients received radiotherapy alone due to old age and comorbidities, while 53 underwent concurrent chemoradiotherapy (CCRT). Chemotherapy was administered using cisplatin at a dose of 40 mg/m^2^ weekly for six cycles during the radiotherapy. RT simulation was performed through a computed tomography (CT) scan of the abdomen and pelvis, with patients in a supine position using a LightSpeed RT 16 CT scanner. The patients performed bladder filling for 2 h before the simulation. On the pre-enhanced simulation scan, if the location of the target lesion was significantly displaced by excessive gas or feces in the rectum, an enema was performed at the discretion of the radiation oncologist. Pelvic EBRT was delivered using the four-field box technique for 38 patients and IMRT for 22 patients. Extended-field RT was applied to eight patients with para-aortic or upper common iliac lymph node metastasis, using the upper border of the T12-L1 spine junction. The median prescribed dose for pelvic RT was 5040 cGy over 28 fractions (range, 4500–5940 cGy/25–33 fractions). A midline block was used for 27 patients after delivering 45 Gy. Due to the lack of brachytherapy facilities at our institution, patients who completed EBRT were referred to three external centers for brachytherapy. All three centers employed high-dose rate ICBT with a remote after-loading system using iridium-192. ICBT was prescribed at point A at a median dose of 28 Gy over 7 fractions (range, 9.6 Gy–32 Gy/3–8 fractions) and administered twice a week. No patients received interstitial brachytherapy. The most prescribed dose of ICBT was 30 Gy over 6 fractions (15 out of 60 patients, 25%), followed by 24 Gy over 6 fractions (14 patients, 23.3%) and 28 Gy over 7 fractions (12 patients, 20%). The median biologically equivalent dose in 2 Gy fractions with a linear–quadratic model using an alpha/beta ratio of 10 (EQD2_10_) was 32.67 Gy_10_ (range, 10.56–40 Gy_10_). Fifty-eight patients underwent two-dimensional brachytherapy (2D brachytherapy) using radiography, and image-guided brachytherapy with CT was performed in two patients. The details of the treatment characteristics are provided in Table 1.

### 2.3. Follow-Up and Toxicity Evaluation

The patients underwent history taking, physical examinations, complete blood counts, and routine chemistry tests during RT at 1-week intervals. After RT completion, follow-up visits were scheduled for 1 month post-RT, then every 3 months for 2 years, followed by every 6 months thereafter. During follow-up, patients underwent history taking and gynecological and imaging evaluations. Magnetic resonance imaging (MRI) of the pelvis was performed at 3-month intervals for 2 years and thereafter at 12-month intervals for 5 years after treatment. CT follow-ups of the chest, abdomen, and pelvis were conducted at 3-month intervals for 2 years and thereafter at 6-month intervals for 5 years.

Late toxicity was defined as toxicity occurring more than 90 days after the completion of RT. Toxicity was evaluated using the criteria of the Radiation Therapy Oncology Group and the European Organization for Research and Treatment of Cancer (RTOG/EORTC) [10] for the lower GI tract and bladder. Toxicity was graded from 0 to 4 at 6, 12, 24, 36, and >36 months after the completion of RT. The onset and prevalence of toxicity at each time point post-RT were investigated. Onset was defined for toxicities not identified in a previous period but newly confirmed during the current period, and prevalence was evaluated as the total toxicity identified at the time of assessment. In cases of hematochezia or hematuria, additional evaluations using endoscopy or cystoscopy were performed. The time of onset from RT completion and the duration of symptoms were recorded. Therapeutic interventions, such as transfusions, endoscopy, cystoscopy, and elective surgery, were investigated, as well as the improvement following such interventions. The clinical remission of toxicity was also evaluated. Complete remission was defined as the complete disappearance of clinical symptoms, and partial remission was defined as a reduction in the grade of toxicity. Grade 4 toxicities, such as fistulas and perforations, were classified as non-remission toxicity due to aspects of their permanent damage. Complications attributed to recurrent cancer were not classified as treatment toxicity.

### 2.4. Statistical Analysis

Progression-free survival (PFS) and overall survival (OS) were defined as the duration between the completion of RT and the occurrence of recurrence, death, or the last follow-up. PFS and OS were estimated using the Kaplan–Meier method.

## 3. Results

The median follow-up duration was 40.9 months (range 12.1–153.1 months). Of the 60 patients, 19 experienced recurrence, and 18 died by the last follow-up. The 5-year PFS and OS rates were 66.9% and 74.7%, respectively. The median age of the patients was 60 years (range 36–84 years). Twenty-two patients received regular cervix cancer screening through pap smears at least at 2-year intervals before the diagnosis of cervix cancer. At the time of diagnosis, 46 patients showed cancer-related symptoms such as vaginal bleeding, discharge, and pain. The predominant histology was squamous cell carcinoma (57 patients, 95%). Adenocarcinoma and adenosquamous cell carcinoma were observed in two and one patient, respectively. At the time of cervical cancer diagnosis, 17 patients were on medication for diabetes mellitus (DM) and 27 for hypertension. The most common FIGO stage at diagnosis was IIB (19 patients, 31.7%), followed by IIIC1 (14 patients, 23.3%) and IVA (8 patients, 13.3%). The clinical characteristics of the patients are detailed in Table 2.

### 3.1. Occurrence of Late Toxicity

Late toxicity was observed in 24 out of 60 patients (40%), with 9 experiencing both lower GI and bladder toxicity. Lower GI toxicity was noted in 18 patients (30%), with ≥grade 2 toxicity occurring in 14 (23.3%). The most common symptom was radiation proctitis accompanied by rectal bleeding (12 out of 60 patients, 20%). Grade 4 GI toxicity included one case of a colovesical fistula and another of a rectovaginal fistula with sigmoid colon perforation. There were no deaths attributed to treatment toxicity. Bladder toxicity was identified in 15 patients (25%), with ≥grade 2 toxicity in 11 (18.3%). The primary symptoms of bladder toxicity included urinary incontinence, frequency, and hematuria, with macroscopic hematuria reported in six patients. Grade 4 toxicity was seen in four patients: one with hemorrhagic cystitis and three with vesicovaginal fistulas. For the patient with hemorrhagic cystitis, hyperbaric oxygen therapy and a neobladder were recommended due to repeated moderate hematuria and limited bladder capacity; however, the patient was subsequently lost to follow-up. A summary of the occurrence of late toxicity is presented in Table 3.

### 3.2. Onset and Prevalence over Time after Completion of Radiotherapy

**Figure 1:**
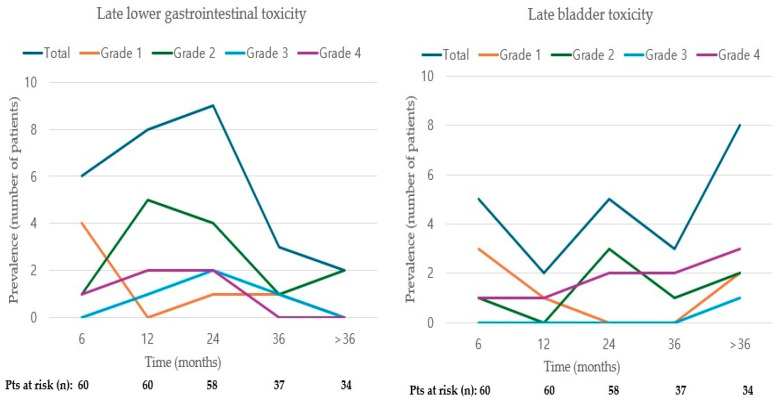
The prevalence of late lower gastrointestinal and bladder toxicity over time following radiotherapy. Pts, patients; n, number.

The median onset time of GI toxicity following the completion of RT was 7.3 months (range, 5.2–58.5 months). For rectal bleeding specifically, the median onset time was 12.7 months (range, 5.2–58.5 months). The median duration of GI toxicity symptoms was 9.9 months (range, 0.1–26.3 months). Ten patients experienced a subsidence of symptoms within one year, five patients within one to two years, and one patient’s symptoms persisted for more than two years. Two patients underwent surgery due to severe and permanent damage from fistulas. The period with the highest onset of GI toxicity occurred 12 months after the completion of RT, with the highest prevalence rate (15.5% or 9 of 58 patients) observed at 24 months. The prevalence rate of GI toxicity at three years post-RT was 8.1% and showed a decreasing trend thereafter (Figure 1). In terms of bladder toxicity, the median onset time post-RT was 20.2 months (range, 31–76.3 months). The median duration of bladder toxicity symptoms was 6.1 months (range, 0.6–13.0 months). Low-grade (grade 1–2) bladder toxicity, which either persisted during RT or appeared immediately afterward, resolved completely or partially within 12 months of completing RT. The first peak onset of bladder toxicity occurred at 24 months post-RT, with occurrences continuing beyond 36 months, indicating a pattern of a re-increasing prevalence rate (23.5%) after 36 months (Figure 1). Of the five cases of grade ≥3 bladder toxicity, three occurred after ≥36 months post-RT. The onset and prevalence of toxicity over time after the completion of RT are detailed in Table 4.

### 3.3. Intervention and Remission of Toxicity

Surgery was performed on two patients who developed significant complications: one with a rectovaginal fistula and sigmoid colon perforation and the other with a colovesical fistula. The procedures performed were Hartmann’s surgery with conversion and anterior resection with partial bladder resection, respectively. Endoscopic intervention was performed in eight patients experiencing rectal bleeding. Argon plasma coagulation (APC) was applied to the bleeding foci, with the average number of APC sessions per patient being 1.5 (range, 1–3 times). The complete remission of rectal bleeding after APC was achieved in seven out of eight patients (87.5%). Transurethral electrocauterization was performed on two patients with hematuria; in one case of severe hemorrhagic cystitis, the hematuria persisted after the intervention. Of the 18 patients who experienced lower GI toxicity, 12 (66.7%) achieved complete remission by the last follow-up date. The evaluation of toxicity remission was limited in one patient due to local recurrence in the affected area. All instances of grade 1 GI toxicity subsided within six months, while 75% of grade 2 and 66.7% of grade 3 toxicities showed complete remission by the last follow-up. In cases of bladder toxicity, 9 out of 15 patients (60%) achieved complete remission. The remission of toxicity according to grade is detailed in Table 5.

## 4. Discussion

We investigated the late toxicities affecting the lower GI tract and bladder in patients with cervical cancer who received pelvic EBRT followed by brachytherapy, reviewing the clinical course and characteristics of these toxicities over time. In this study, the late toxicity of the lower GI tract and bladder occurred in 30% and 25% of patients, respectively. These rates are consistent with those reported in previous studies of definitive RT for cervical cancer, where rectal toxicity ranged from 29.7% to 40% and bladder toxicity from 21.8% to 28% [6,11,12]. The most common late GI toxicity observed was rectal bleeding, aligning with findings from a previous study [12]. Regarding the clinical course of late GI toxicity, the onset and remission of most late GI toxicities were shown within three years of completing RT, under efficient management strategies such as APC. These findings are consistent with those of a previous study by P. Georg et al., which analyzed the prevalence rates of late toxicity in patients with cervical cancer treated with definitive RT [12]. In a study by P. Georg et al., the mean time to the onset of rectal toxicity was 14 ± 8.5 months, with all late rectal toxicity occurring within three years after RT. A total of 81% of patients who developed rectal toxicity showed remission within three years of onset, and the actuarial prevalence rate at three years post-RT was 9%, consistent with the results of this study (8.1% at three years). Conversely, bladder toxicity first peaked at 24 months post-RT and continued to occur beyond 36 months, demonstrating a pattern of re-increasing prevalence (23.5%) after 36 months. In this study, six cases of bladder toxicity occurred at ≥36 months post-RT, three of which were ≥grade 3. These results suggest that severe bladder toxicity is likely to occur even more than three years after RT. The latent period for urologic complications post-RT has been reported to extend up to 30 years, and the risk of developing adverse events increases over time [13,14]. According to the study by P. Georg et al., the prevalence rates of late bladder toxicity at three and five years post-definitive RT for cervical cancer were 18% and 21%, respectively. Unlike late GI toxicity, bladder toxicity can occur long-term after RT completion, with nearly 20% of patients experiencing toxicity between three and five years post-RT. This suggests the need for closer follow-up for late bladder toxicity after definitive RT for cervical cancer over a long-term period even after five years.

For the evaluation of the clinical remission of late toxicities, 66.7% of late GI toxicity cases showed complete clinical remission, and 11.1% showed partial remission by the last follow-up period. In cases of rectal bleeding, 87.5% achieved complete remission after treatment with APC, demonstrating its therapeutic effectiveness. Previous studies also confirm the efficacy and safety of APC for rectal bleeding, with success rates ranging from 87% to 91.5% [15,16]. In cases of late bladder toxicity, grade 1-2 toxicities typically followed a favorable course, with all showing complete or partial remission. However, ≥grade 3 toxicities often presented a chronic course, characterized by repetitive gross hematuria or permanent damage such as vesicovaginal fistulas. Currently, for severe radiation cystitis, treatments including systemic therapy with sodium pentosan polysulfate, cystoscopy fulguration or electrocoagulation, and hyperbaric oxygen therapy (HBOT) are employed [17,18]. The effectiveness of HBOT for radiation cystitis has been reported with varying success rates of 72.7–92.4% [19,20,21,22]. The study by Chang et al. [22] noted that 96% of patients showed improvement in hematuria when HBOT was administered within six months from the onset of hematuria. It has been reported that the early diagnosis and treatment of radiation cystitis significantly increase the chances of complete healing [18]. In the follow-up of cervical cancer patients who underwent definitive RT, it may be crucial to maintain close observation for the occurrence of radiation cystitis over a long-term period and initiate timely therapeutic interventions based on the grade of radiation cystitis.

There are several limitations to this study. First, due to the relatively small cohort size, the occurrence of events might have been limited, and some toxicities could have been underestimated due to the nature of this retrospective study. Second, the use of image-guided brachytherapy, which is the recommended RT technique for cervical cancer, was limited; only two patients underwent CT-guided brachytherapy, which could have increased the occurrence of overall late toxicity. According to the current state of brachytherapy in Korea, only 48.2% and 37.9% of centers perform image-guided brachytherapy and volume-based planning, respectively, due to limitations in brachytherapy facilities and human resources [23]. However, this study identified specific clinical courses and characteristics of late toxicity in patients with cervical cancer who underwent combined EBRT and brachytherapy by analyzing the onset and prevalence of late toxicity at each time point after the completion of RT. Additionally, by presenting the distinct characteristics between late GI and bladder toxicity, it provides information for a more efficient strategy for the follow-up and management of late toxicity.

## 5. Conclusions

The late toxicities of the GI tract and bladder in definitive RT for cervical cancer are partially reversible and exhibit distinct characteristics in terms of onset and prevalence over time. Based on an understanding of these clinical courses and characteristics, a more structured follow-up strategy needs to be established to facilitate early diagnosis and intervention for late toxicity.

## Figures and Tables

**Table 1:** Treatment’s characteristics.

Variable	*n*. (%), Total = 60
Modality	
Concurrent chemoradiotherapy	53 (88.3%)
RT alone	7 (11.7%)
EBRT dose	Median, 50.4 Gy/28 fxs (range, 45–59.4 Gy/25–33 fxs)
EBRT technique	
4-box technique	38 (63.3%)
IMRT	22 (36.7%)
Brachytherapy prescription	
9.6 Gy/3 fxs	1 (1.7%)
19 Gy/4 fxs	1 (1.7%)
20–22.5 Gy/5 fxs	3 (5%)
24 Gy/6 fxs	14 (23.3%)
28 Gy/7 fxs	12 (20%)
32 Gy/8 fxs	3 (5%)
25–27.5 Gy/5–6 fxs	8 (13.3%)
30 Gy/5 fxs	3 (5%)
30 Gy/6 fxs	15 (25%)
Cumulative EQD2_10_ to point A	Median, 82.23 Gy_10_ (range, 60.12–95.3 Gy_10_)
<80 Gy_10_	20 (33.3%)
≥80 Gy_10_	40 (66.7%)

*n*., number; RT, radiotherapy; EBRT, external beam radiation therapy; fxs, fractions; IMRT, intensity-modulated radiation therapy; EQD2_10_, biologically equivalent dose in 2 Gy fractions with a linear–quadratic model using an alpha/beta ratio of 10.

**Table 2:** Patient characteristics.

Variable	*n*. (%), Total = 60
Age	Median, 60 years (range, 36–84 years)
≤45 years	4 (6.7%)
>45 years	56 (93.3%)
Menopausal status at diagnosis	
Premenopause	11 (18.3%)
Menopause	49 (81.7%)
Regular cervix cancer screening before diagnosis †	
Yes	22 (36.7%)
No	38 (63.3%)
Symptom due to cervix cancer at diagnosis	
None	14 (23.3%)
Vaginal bleeding	33 (55%)
Vaginal discharge	5 (8.3%)
Pain	7 (11.7%)
Hydronephrosis	1 (1.7%)
Diabetes mellitus	
Yes	13 (21.7%)
No	47 (78.3%)
Hypertension	
Yes	23 (38.3%)
No	37 (61.7%)
Smoking	
Smoker	5 (8.3%)
Non-smoker	55 (91.7%)
Pathology	
Squamous cell carcinoma	57 (95%)
Adenocarcinoma	2 (3.3%)
Adenosquamous carcinoma	1 (1.7%)
Tumor size (maximum diameter)	Median, 4.6 cm (range, 1–10 cm)
Pre-treatment SCC Ag	Median, 7.5 (range, 0.55–88.2)
LN metastasis	
Yes	24 (40%)
No	36 (60%)
FIGO stage	
IB	9 (15%)
IIA	4 (6.7%)
IIB	19 (31.7%)
IIIB	3 (5%)
IIIC1	14 (23.3%)
IIIC2	3 (5%)
IVA	8 (13.3%)

*n*., number; SCC Ag, squamous cell carcinoma related antigen; LN, lymph node; FIGO, International Federation of Gynecology and Obstetrics. † Screening was performed through regular pap smears at least at a 2-year interval.

**Table 3:** Occurrence and manifestations of late toxicity.

Late Rectal Toxicity	*N* of pts (%)	Manifestations
G1	4 (6.7%)	diarrhea (*n* = 4)
G2	9 (15%)	rectal bleeding (*n* = 9)
G3	3 (5%)	moderate rectal bleeding (*n* = 3)
G4	2 (3.3%)	rectovaginal fistula (*n* = 1),
		colovesical fistula (*n* = 1)
Late Bladder Toxicity		
G1	4 (6.7%)	urinary incontinence (*n* = 1),
		dysuria (*n* = 1),
		urinary frequency (*n* = 2)
G2	6 (10%)	urinary incontinence (*n* = 1),
		urinary frequency (*n* = 1),
		gross hematuria (*n* = 4)
G3	1 (1.7%)	moderate hematuria (*n* = 1)
G4	4 (6.7%)	hemorrhagic cystitis (*n* = 1),
		colovesical fistula (*n* = 1),
		vesicovaginal fistula (*n* = 2)

*N*, number; pts, patients.

**Table 4:** The onset and prevalence of late toxicity over time following radiotherapy.

Toxicity	Number of Patients									
	3–6 Months		12 Months		24 Months		36 Months		>36 Months	
	New †	P. ‡	New	P.	New	P.	New	P.	New	P.
Rectum										
Gr 1	4	4	0	0	0	1	0	1	0	0
Gr 2	1	1	4	5	2	4	1	1	1	2
Gr 3	0	0	1	1	1	2	0	1	0	0
Gr 4	1	1	1	2	0	2	0	0	0	0
Total	**6**	**6**	**6**	**8**	**3**	**9**	**1**	**3**	**1**	**2**
Bladder										
Gr 1	3	3	0	1	0	0	0	0	1	2
Gr 2	1	1	0	0	3	3	0	1	2	2
Gr 3	0	0	0	0	0	0	0	0	1	1
Gr 4	1	1	0	1	1	2	1	2	1	3
Total	**5**	**5**	**0**	**2**	**4**	**5**	**1**	**3**	**5**	**8**
pts.at risk * †	60		60		58		37		34 **	

P., prevalence; Gr, grade; N., number; pts., patients. †, number of newly confirmed events at a relevant time. ‡, prevalence; number of total patients showing toxicity at a relevant time. * Number of patients at risk. ** Exclusion of 26 patients: 3 patients who did not reach 36 months after the completion of radiotherapy, 11 who died before the relevant period, and 12 who were lost to follow-up.

**Table 5:** Remission of late toxicity according to grade of toxicity.

Toxicity	Complete Remission *n*. (%)	Partial Remission *n.* (%)	Non-Remission *n.* (%)
Lower GI †			
Grade 1	4/4 (100%)	0/4 (0%)	0/4 (0%)
Grade 2	6/8 (75%)	1/8 (12.5%)	1/8 (12.5%)
Grade 3	2/3 (66.7%)	1/3 (33.3%)	0/0 (0%)
Grade 4	Fistula and perforation (*n.*= 2); permanent damage		
Bladder			
Grade 1	4/4 (100%)	0/4 (0%)	0/4 (0%)
Grade 2	5/6 (83.3%)	1/6 (16.7%)	0/6 (0%)
Grade 3	0/1 (0%)	0/1 (0%)	1/1 (100%)
Grade 4	Fistula and hemorrhagic cystitis (*n.*= 4); permanent damage		

*n*., number; GI, gastrointestinal. † The remission of gastrointestinal toxicity was evaluated in a total of 17 patients, excluding 1 patient who subsequently developed recurrence in the affected area of toxicity.

## Data Availability

Data are available upon reasonable request.
